# Address to the Graduating Class of Geneva Medical College

**Published:** 1865-01

**Authors:** Wm. D. Wilson


ART. II.—*Address to the Graduating Class of Geneva Medical College,*
BY PROF. WM. D. WILSON, M. D.

PEEKSKILL, Dec. 21, 1864.

*Editor Buffalo Medical & Surgical Journal:*

DEAR SIR: The enclosed Address has been kindly presented to me by the author, to dispose of as I should deem proper. On perusal, I find its views so liberal and just, that I can not but think great good might result from its publication. If you agree with me in this opinion, you may confer a favor upon your readers by finding a place for it in the columns of your valuable Journal.

CHARLES A. LEE, M. D.

YOUNG GENTLEMEN: In the absence of Dr. Hale, it becomes my duty and my pleasure to confer upon you the honorable distinction of Doctor in Medicine. Nor is it proper that an act of so much importance in many respects should be suffered to pass as a mere formality. Such distinctions are conferred for the purpose of indicating to the community, who naturally look to those more experienced and wiser than themselves—those persons with whom they may safely entrust the important interests which the learned professions were designed to subserve. With the degree which we are now about to confer upon you, you go forth to the world with our certificate and recommendation. We endorse your qualtifications as Medical Practitioners. We have a right, therefore, not only to give you the instructions which pertain to the theory and practice of your profession, but also to insist somewhat emphatically upon the dignity and the responsibilities of the honorable distinction which we confer upon you.

You commence your lives and your labors, young gentlemen, at a period in the world's history which is peculiarly interesting and peculiarly trying. Never was quackery, in all forms, more prevalent. Never were the results of accumulated wisdom and experience so lightly regarded as now. And you go into the world, not merely as practitioners of medicine—not merely to pursue a trade which you have learned as a means to wealth and self-aggrandizement. You go out as members of one of the learned professions. You go forth to exert an influence beyond the mere province of the art of healing. You have duties and responsibilities reaching far beyond the mere business maxims of doing so much work for so much pay. You form an important element in that portion of our country on which we must rely for a safeguard against the extravagances of fanaticism, imposture and licentiousness.

I have said that quackery, in all its forms, is prevalent—never so prevalent as now. It is a mistake to suppose that it is confined to the practice of medicine alone. As educated physicians you know that there are some diseases that are incurable, and some stages in all diseases that put them beyond the reach of any efficient treatment; and that in others relief and cure can be accomplished only by time and slowly acting remedies. The same is true in the body politic, and in the church—in every department of human society. But human nature, impatient of the infirmities and slow processes incident to everything human, is ready to listen to the quack who comes with the promise of what the man of science knows cannot be accomplished, and what the man of integrity will not promise.

This is not confined to bodily ailments alone. The soul of man is not less diseased than his body. Hence in the church and in the state there is a field for quackery, and unfortunately there are men enough to avail themselves of the opportunity presented. Some new and easy method is devised, and as a plea in excuse for its adoption and as a means of gaining distinction for its invention a new theory is promulgated, new schools and sects are founded.

We are not however to pass these experiments by, as unworthy of attention, or as things which are to be noticed only to be condemned. They deserve our serious study, and chiefly in two respects.

1.—As containing in themselves, each of them, some element of truth, they often serve to point out an error in the prevalent theory and practice. No theory or sect can live long which contains no element of truth, and is not designed as a remedy for something which is really felt to be an evil. I do not say that the appearance of a new theory or a new sect *always* proves that the old one was wrong, in the point of divergence and contrariety, between it and the new. But still, this is so often the case, that each new theory, however wild, deserves to be considered with reference to this point.

In medicine I suppose it will be conceded that the theory which prevailed when Homœopathy made its appearance was erroneous in the direction of depleting too much and in over-dosing the patient. Homœopathy was practically the opposite—the other pole of the true theory. The same holds true in religion and in politics. Let a church become formal and superstitious, and a party will be formed, as at the Reformation to deny all use of formularies and ceremonies, and to condemn all reliance on public authority in matters of faith. Let this party become a church by itself, and err, as it is sure to do in the direction in which it first started off, and a reaction will take place—a party will rise within its own communion advocating to an extravagant degree the sacredness of church authority and the necessity of ceremonies and formularies.

In politics the same thing will occur. The extreme of absolutism will produce a party of Red Republicans, and out of these same Red Republicans will arise a party in the course of time which will advocate monarchy and even acquiesce in despotism, as preferable in the contrast, to the licentiousness and anarchy which result from too much of liberty.

Thus it is, gentlemen, whether it be in matters relating to the body physical or the body politic, or in matters of religion, each new theory or school is a hint for us. We should first understand precisely what it is, and what it aims to accomplish, and then reconsider the received theory on this point. In this way a large part of the great steps in the advancement of science and the work of reformation has been brought about. The new school or sect for the most part dwindles to insignificancy and extinction so soon as the advocates of the received system fully apprehend and adopt the truth it started upon, I may indeed say that this is always the case, except when the old system or church forfeits its right to existence by blindly and obstinately adhering to its errors. Such seems to be the law and order of Providence.

2.—But secondly, each new school, sect or system is to be observed and studied as an experiment on a large scale. It is putting to the test of experience not only the new theory, but also (as a necessity of the case) its counterpart—the old. Undoubtedly the maxim is, "always adhere to the old until something new has been proved to be better." And yet if *all* persons were to adhere to this maxim "*the new*" in most cases never could be "*proved to be better*," since no one would test it by experience. But while others show no reluctance to try these experiments, whose result is at best but doubtful, we may well content ourselves to stand by and look on. If the experiment succeeds it enlarges the sphere of our knowledge; it augments our resources and may require some modification of that which we had previously held as indubitable truth. But if it fails, and history shows that by far the greater proportion of them will fail, it only confirms the wisdom of that which had already stood the test of experience, and although perhaps not perfect, (nothing human is so,) yet was *well.*

There is, gentlemen, an ethical principle involved in this matter which I fear is not appreciated so highly as it ought to be. To illustrate it let me give you an instance: If a person dependent upon me is sick, and it devolves upon me to procure medical treatment, it is not to be supposed that I—not having been educated in the medical profession—am competent to judge of medical theories and modes of treatment. If I were I should not need to call in the aid of others. But how am I to determine whom I shall call? I am responsible for the life or death of a wife or child perhaps to a large extent. How am I to meet that responsibility? Is it enough that I call in one whom, in my caprice or my ignorance, I may have acknowledged to be a safe practitioner? I apprehend not. Doubtless I am bound to call in the best aid that I can get. And in all cases every principle of wisdom, of experience, of ethics, and, we may add, of human law, also teaches that that is to be considered "the best aid" which, while professing to receive and adopt all new discoveries and improvements so soon as they shall have been sufficiently tested and established, does yet and nevertheless profess to retain and hold fast all that has been communicated in the past.

And what is true in principle for those who may have occasion to call in medical advice and aid must be true with the proper modifications for you who may be called to administer. Certain and unfailing success is not within the reach of any man. But to act in such a way as to be free from responsibility for the result, and free from guilt if a result be adverse to our wishes, is within the reach of all. And the simple maxim guiding to it is this: "*Do in all cases that which the best wisdom and experience of the past has shown to be the safest and surest method.*" Experiments involving *totally* new principles and theories *may succeed*, but they are never safe. Nor are they ever an excuse for failure if failure ensues.

I claim no infallibility for this method. It may fail, and the result be most disastrous to the dearest interests of man. This is only a misfortune incident to, and inseparable from, all human affairs. But still this is the wisest course. It is the only course that meets our responsibilities and satisfies the demands of our obligations to act as wise and rational beings, in the fear of God, and with due regard to the welfare of our fellow men. We cannot always wait to judge by results. We must have some rule or principle to guide us before hand. The results often do not appear until it has become too late to choose between the different modes of practice.

But such is the law of Providence everywhere and in every department of human thought and human science. Wisdom accumulates by experience; and progress is always most wisely sought and most surely found there where the treasured results of past experience are both carefully and professedly retained. There shine forth genius and wisdom.—There gratitude for the labors of past ages, is not extinguished by the ignorance and conceitedness of the present. Into this onward flowing stream every rill and rivulet of truth is sure at last to flow, and become mingled and absorbed in its ever widening channel and ever accumulating volume.

You are not to understand by these remarks however that no progress is to be made in your profession—no experiments to be tried. On the contrary progress is a duty; there is a legitimate sphere of experiment within the current of accumulated wisdom. Cases will arise in which after the old method has been tried and failed, something new is demanded, There will also be cases for which there is no established prescription. And here is the field for experiment and improvement. But these are not the experiments of what I have been speaking. They repudiate the past as a mass of error and superstition. They start on theories totally new, and disclaim the only law of true progress by which each age, instead of beginning anew, starts with the accumulated wisdom of the past.

Now, gentlemen, you may, if you choose, (it is certainly in your power to do so,) degrade the dignity of your profession, each in his own case, to the level of a mere trade—a handicraft whereby to procure a livelihood. But you can never put yourselves in the position of other men. You can never escape the responsibilities of your position. Your very profession, and the testimonials which we this day confer upon you, put you into a respectable and an influential position in society. An opinion expressed or a word uttered by you will have a wider sphere of influence and fall with a heavier weight of authority than if it came from one who had not had your opportunities, and who did not hold in his hand the high testimonials and sanctions with which you go forth to your work. And your accountability to God and to man is proportionately great. An immoral sentiment, an infidel opinion, nay, a word of levity or jeering on sacred subjects, coming from you, will endanger souls for time and for eternity. The course of study you have pursued, and the experience with which you will meet in the practice of your profession, ought to make you sober, thoughtful and pious men. And if there be a man on the face of the earth whom, next after the parish minister, all men we would have to be a sober, God-fearing man, it is the family physician. Let me request you to add to these influences those which will result from a frequent and faithful consideration of the responsibilities of your position, and the dread account you will have to render at the last day, when each must give account of himself unto God for the deeds done in the body.

Pursuing this course you cannot fail to be respected everywhere, and to be rewarded with the confidence and gratitude of those among whom your lot in life may be cast. And the practice of your profession, if it do not make you rich, will bring you what is better than wealth, a recompense more imperishable and more satisfying than any earthly substance you can acquire.

Nor can you in this way fail to add something to the stock of knowledge and experience of one of the noblest professions. Not by rash experiment, bold assumption, or wild theories, but by thoughtful, sober, cautious attention to each case that may come before you will you best perfect your own characters and knowledge as medical practitioners, and best advance the honor of your profession and conduce most successfully to the benevolent work of alleviating human suffering.

